# Patterning and Development of Photoreceptors in the Human Retina

**DOI:** 10.3389/fcell.2022.878350

**Published:** 2022-04-14

**Authors:** Katarzyna A. Hussey, Sarah E. Hadyniak, Robert J. Johnston

**Affiliations:** Department of Biology, Johns Hopkins University, Baltimore, MD, United States

**Keywords:** retina, photoreceptor, cone, rod, human, macula, thyroid hormone, retinoic acid

## Abstract

Humans rely on visual cues to navigate the world around them. Vision begins with the detection of light by photoreceptor cells in the retina, a light-sensitive tissue located at the back of the eye. Photoreceptor types are defined by morphology, gene expression, light sensitivity, and function. Rod photoreceptors function in low-light vision and motion detection, and cone photoreceptors are responsible for high-acuity daytime and trichromatic color vision. In this review, we discuss the generation, development, and patterning of photoreceptors in the human retina. We describe our current understanding of how photoreceptors are patterned in concentric regions. We conclude with insights into mechanisms of photoreceptor differentiation drawn from studies of model organisms and human retinal organoids.

## Introduction

**FIGURE 1:**
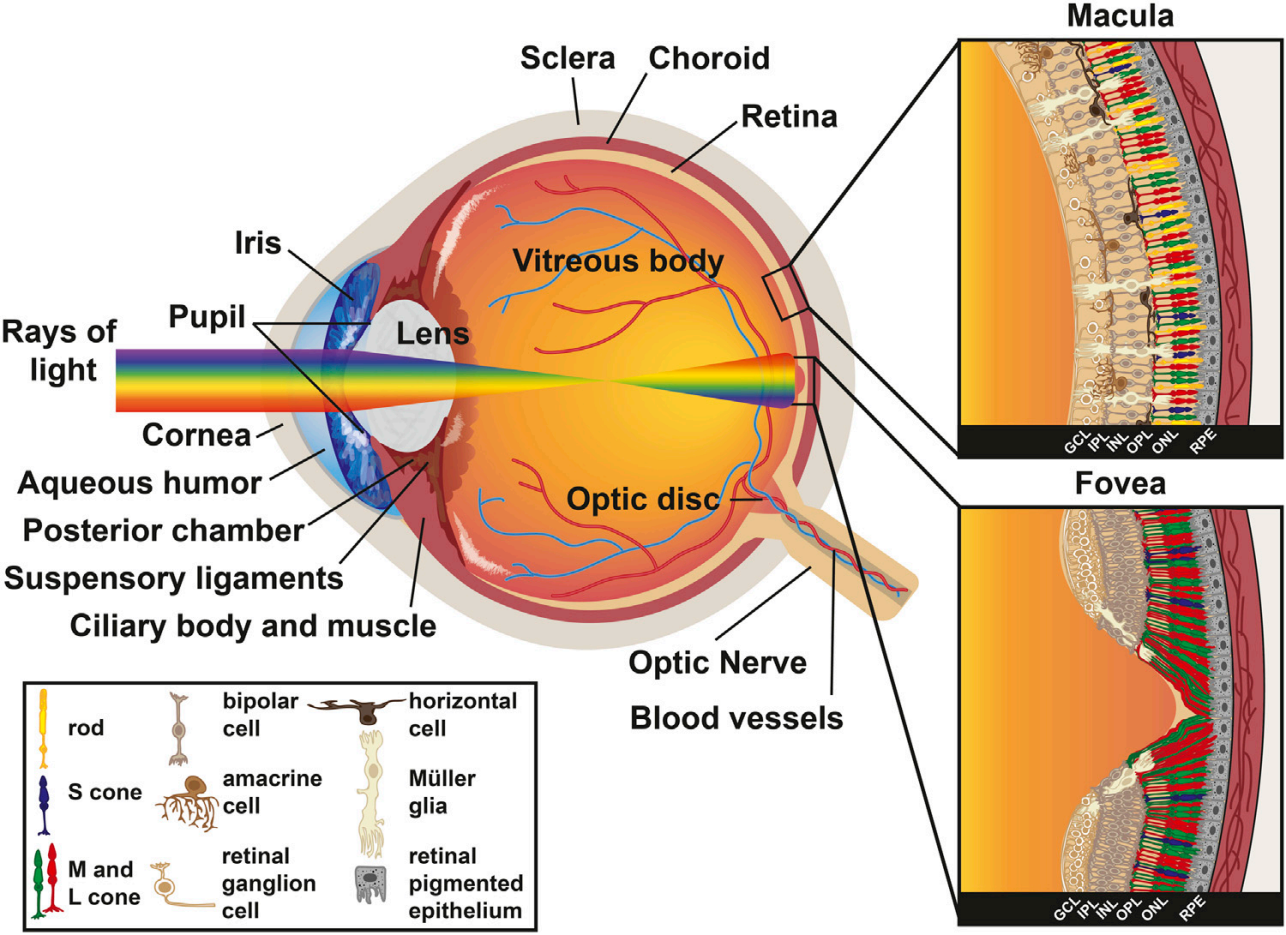
Anatomy of the human eye. Diagram of a human eye with the light path represented as a rainbow. Inset sections represent the retinal cell types present in the macula (top) and the fovea (bottom). Key describes retinal cell types.

Humans and other primates use their sense of sight as a primary mechanism for navigating their environments. The human camera eye relies on the cornea and lens to focus light onto the retina, a tissue located in the back of the eye. Vision begins with the detection of light by photoreceptor cells within the retina (Figure 1).

Unlike other placental mammals, primates have trichromatic color vision and high visual acuity. Trichromacy is achieved by three distinct cone photoreceptor subtypes that enable the sensation of blue, green, and red wavelengths of light. The three cones signal through interneurons and retinal ganglion cells to the brain to perceive the colors of the visible spectrum.

The goal of this review is to describe patterning and development of the photoreceptors in the human retina. We briefly describe the major cell types of the human retina and their roles in vision. We then focus on photoreceptors, discussing how light sensitivities, cell morphologies, gene expression profiles, and functions define photoreceptor subtypes. Next, we discuss how genetic and signaling pathways influence photoreceptor subtype specification. We continue with the patterning of photoreceptor subtypes across the retina, with a particular focus on the fovea and foveola, the structures that enable high visual acuity in primates. We conclude with a description of how studies of human retinal organoids have advanced our understanding of photoreceptor subtype differentiation and how organoids serve as a promising new model to understand mechanisms of retinal development and disease.

## Retinal Cell Types

The vertebrate eye contains five major neuronal types generated from a common progenitor pool: photoreceptors, bipolar cells, retinal ganglion cells (RGCs), horizontal cells, and amacrine cells. Retinal progenitor cells also generate one glial type called Müller glia. The cell types of the retina are stratified into three layers of cell bodies and two plexiform layers: 1) the outer nuclear layer (ONL) contains the photoreceptors, 2) the outer plexiform layer (OPL) contains the synapses from the photoreceptors onto interneurons, 3) the inner nuclear layer (INL) contains the cell bodies of bipolar, horizontal, and amacrine interneurons, which transmit and process visual information, and Müller glia, which provide structural support and maintain the extracellular environment, 4) the inner plexiform layer (IPL) is the location where interneurons synapse onto the retinal ganglion cells, and 5) the ganglion cell layer (GCL) primarily contains retinal ganglion cells that relay information from the eye to the brain (Figure 1).

The layers of the human retina are inverted relative to the path of light. Light passes through the GCL first, continues through the IPL, INL, and OPL, and is absorbed by photoreceptors whose nuclei lie in the ONL (Figure 1). Müller glia act as light cables that guide the yellow-green portion of the visible light spectrum (around 560 nm) through the RGCs and interneurons, directly to cone cells (Franze et al., 2007; Agte et al., 2011; Labin et al., 2014; Agte et al., 2018). Müller glia also play important roles in their interactions with photoreceptors and other neurons, acting as sinks for metabolic waste and removal of excess neurotransmitter within the retina (Newman, 1994; Tsacopoulos and Magistretti, 1996). Stray photons are absorbed in the retinal pigmented epithelium (RPE), a melanin-rich epithelial cell layer that lies behind the retina. The RPE recycles retinal, a light-sensitive chromophore required for the detection of light by the opsin protein, from all-*trans*-retinal into 11-*cis*-retinal and returns it to photoreceptors, which is essential for photoreceptor health and function (Baehr et al., 2003; Thompson and Gal, 2003). Additionally, Müller glia recycle all-*trans*-retinal back to 11-*cis*-retinal for cones exclusively (Mata et al., 2002; Kolesnikov et al., 2021). The RPE also plays a role in phagocytosis of photoreceptor outer segments as they are shed (Strauss et al., 1998; Gal et al., 2000; Finnemann, 2003). Astrocytes, another glial cell type that migrates into the retina via the optic nerve, are also present in the GCL (Stone et al., 1995).

Light information is detected when a photon is captured by a photoreceptor in the ONL. Electrical signals are transmitted back towards the inner retinal layers by bipolar cells, horizontal cells, and amacrine cells in the INL, and conveyed to RGCs in the GCL. Finally, this information is carried by RGCs to the brain.

Photoreceptors, the focus of this review, are classified as either rods or cones and have cell bodies in the ONL (Figure 1). These cell types can be distinguished by their morphologies and spectral sensitivities. Rods, with the sensitivity of single-photon detection, are the cell type responsible for low light vision and motion detection (Baylor et al., 1979). In contrast, cones are primarily involved in high-acuity daytime and color vision (Nathans et al., 1986b; Jacobs and Deegan, 1999). There is estimated to be 92 million rods (77.9–107.3 million) and 4.6 million cones (4.08–5.29 million) in the human retina (Curcio et al., 1990). In humans, the three cone subtypes are defined by the expression of opsin proteins that are optimally sensitive to different wavelengths of light. Cones contain either short-wavelength sensitive opsins (S/blue cones), medium-wavelength sensitive opsins (M/green cones), or long-wavelength sensitive opsins (L/red cones). As S cones comprise the minority (8–12%) of the cone population (Curcio et al., 1991), spatial information appears to be primarily processed by M and L cones (Lennie et al., 1993). We revisit the differentiation and patterning of photoreceptors in the human retina in much greater detail later in this review.

## Photoreceptor Spectral Sensitivities, Morphologies, and Orientations

Photoreceptors are classified based on their expression of light-detecting opsin photopigments, morphologies, functions, and locations in the human retina. Light detection depends on the pathway that light takes when entering and traveling through the eye, as well as the spectral sensitivities of the photopigments expressed in rods and cones. Phototransduction begins as light enters the cornea, the transparent tissue at the most anterior region of the eye. Light then passes through the pupil. Pupil size is regulated by the iris, the pigmented part of the eye, to modulate the amount of light passing into the eye. Light then hits the lens, an ellipsoid, biconvex structure that refracts focused light onto the retina. After the lens, light passes through the aqueous humor. Ciliary muscle contraction regulates the flow of aqueous humor and lens shape. Finally, light reaches the retina and is detected by the photoreceptors (Figure 1).

**FIGURE 2:**
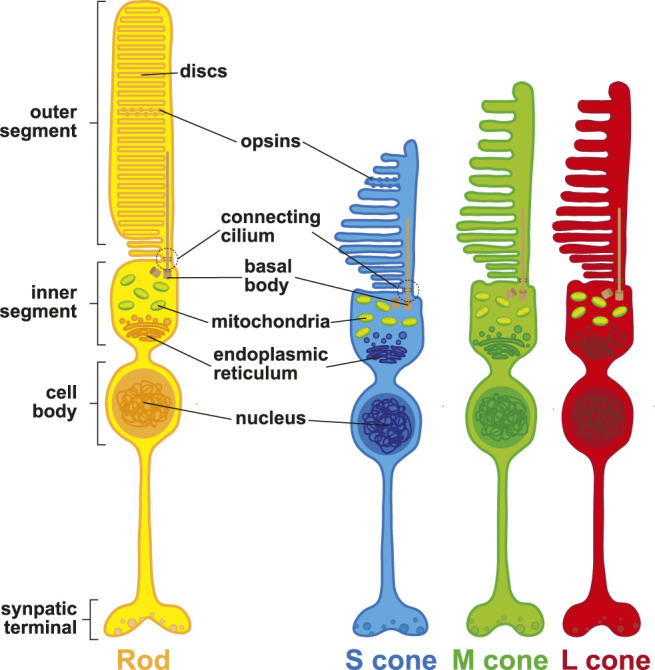
Photoreceptor morphology. Schematic representation of rod photoreceptors (yellow), S (blue), M (green), and L (red) cone photoreceptors.

In order to initiate the phototransduction cascade, photons must pass through the cell bodies of many cell types, including the photoreceptors themselves, before hitting the opsin photopigments in the photoreceptor outer segments (Figure 2). Opsin photopigments are light-sensitive G-protein coupled receptors that are covalently linked to an 11-*cis*-retinal chromophore through a Schiff base (Nathans et al., 1986b; Kosower, 1988; Sakmar and Khorana, 1988; Zhukovsky and Oprian, 1989). Absorption of a photon by the chromophore induces a conformational change to all-*trans*-retinal, which triggers the opsin to initiate the phototransduction cascade (Hubbard and Kropf, 1958). The opsin then activates transducin, a G-protein. Transducin subsequently activates PDE6, a cGMP-phosphodiesterase, which is responsible for conversion of cGMP to GMP, and sodium channels subsequently close. The decrease in the sodium current results in the release of glutamate at the synapse which signals to downstream interneurons (Grossniklaus et al., 2015). In this section, we describe how opsin expression and morphological differences among rods and cones drive functional properties of these photoreceptors.

### Spectral Sensitivities of Opsin Proteins

**FIGURE 3:**
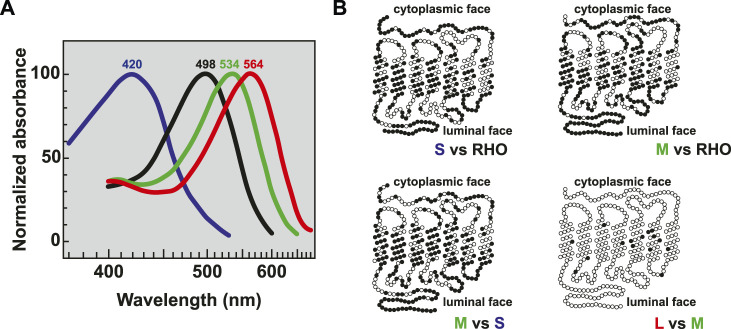
Opsin absorption and comparative morphology. **(A)** Relative absorption spectra of opsins with labeled peaks for S cones (blue, 420 nm), Rods (black, 498 nm), M cones (green, 534 nm), and L cones (red, 564 nm) adapted from (Bowmaker et al., 1980). **(B)** Pairwise comparisons of amino acids between the visual opsin proteins adapted from (Nathans et al., 1986b). Dark circles indicate amino acid differences. Sequences were optimally aligned such that no insertions or deletions were required except for comparing the carboxy termini between the green or red and blue pigments.

The spectral sensitivity of each photoreceptor type is determined by the expression of distinct opsin proteins. Rods express rhodopsin (RHO) and cones express S-, M-, or L-opsin (OPN1SW/MW/LW). The unique spectral sensitivities of the opsins are related to differences in their protein structures, and consequentially their interaction with the light-sensitive chromophore. In humans, absorption maxima are 498 nm for rhodopsin, 420 nm for S-opsin, 534 nm for M-opsin, and 564 nm for L-opsin (Bowmaker et al., 1980) (Figure 3A). Rhodopsin is distinct from the cone opsins, sharing only 42% protein identity with S-opsin, 40% protein identity with L-opsin, and 41% protein identity with M-opsin. S-opsin shares 43% protein identity to L-opsin and 44% protein identity to M-opsin. In contrast, M- and L-opsin are highly similar, sharing 96% protein identity (Francois, 1958; Nathans et al., 1986a; Nathans et al., 1986b) with only 7 nonsynonymous amino acid changes among 20 DNA base pairs that differ between the coding sequence (Nathans et al., 1986b) (Figure 3B). Based on the differences in opsin sequence, immunohistochemistry against opsin proteins can be used to visually distinguish rhodopsin in rods, S-opsin in S cones, and M- or L-opsin in M or L cones. The generation of antibodies specific to M- and L-opsins has been hindered by the very high sequence homology (Szél et al., 1988; Lerea et al., 1989; Wang et al., 1992; Chiu and Nathans, 1994).

### Photoreceptor Morphologies

At their distal end, photoreceptor cells contain an outer segment (Figure 2). The outer segment is a modified cilium that contains the light-detecting opsin photopigments and contacts the extended processes of the RPE. The outer segment is connected through a cilium to an inner segment, which is rich in mitochondria and acts as the site of photopigment and membrane disc synthesis. These discs renew in the outer segments, which are shed over the lifetime of the photoreceptor (Young, 1967). The inner segment is connected to the cell body, which contains the cell nucleus, and lastly axons which project medially into the retina.

Rods, S cones, and M/L cones are distinguished by unique morphological characteristics. Differences in the sizes and shapes of inner segments affect how light is guided through the cell to reach the outer segment (Winston, 1981). Rods have long, cylindrical inner and outer segments. Rod outer segments are stacked with parallel membranous discs independent from the plasma membrane (Sjostrand, 1953). Rod discs have unique protein compositions compared to the plasma membrane, but rhodopsin is found on both membranes (Molday and Molday, 1987). Rod inner segments can be separated into the ellipsoid, which contains a large number of mitochondria, and the myoid, which connects the inner segment to the nucleus. mRNAs for outer segment proteins are localized to the inner segment (Wensel et al., 2016).

In contrast, cones have a characteristic conical shape with tapered ends, and contain parallel membranous discs that are nearly continuous with the plasma membrane (Cohen, 1970). S cones have shorter outer segments than M/L cones. The inner segments of S cones are 10% taller than M/L cones, and their outer segments are wider relative to M/L cones (Ahnelt et al., 1987; Ahnelt et al., 1990; Curcio et al., 1991). M cones and L cones are morphologically indistinguishable from one another. Cone inner and outer segments mature and continue to develop postnatally. Inner segments begin to develop around 25 weeks of gestation. In contrast, outer segments in the fovea do not begin development until 1 week after birth and mature for years until they reach a length of ∼60 µm (Yuodelis and Hendrickson, 1986).

### Photoreceptor Orientation

Nearly a century ago, Stiles and Crawford used psychophysics to show that human eyes are more sensitive to light that enters through the center of the pupil than light entering at the pupil’s edge (Crawford, 1933). Photoreceptors are oriented in the retina such that their outer segments are pointed toward the pupil to enhance light absorption and improve visual acuity in a coordinated alignment of polarized cells (Housset et al., 2021; Verschueren et al., 2022). This orientation is more pronounced in the peripheral retina (Laties et al., 1968; Laties, 1969; Laties and Enoch, 1971; Enoch, 1972). Toward the periphery, the angle between a cone cell body and its outer segment can be as great as 40° (Laties, 1969). All photoreceptors are oriented based on position, though the tilting is more significant in cones, perhaps due to the decreased sensitivity of cone cells compared to rods (Ingram et al., 2016).

## Photoreceptor Subtype Specification

**FIGURE 4:**
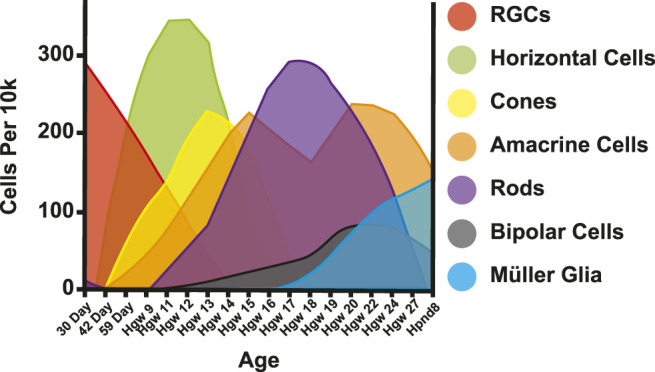
Retinal cell birth order. Adapted from (Lu et al., 2020). Cells per 10k = expected cells in 10k of cells. Day = Day post conception. Hgw = Human gestation week. Hpnd = Human postnatal day.

Multipotent progenitor cells in the retina give rise to different retinal cell types, including photoreceptors (Turner and Cepko, 1987; Wetts and Fraser, 1988; Turner et al., 1990). Cell types are born in conserved, overlapping spatiotemporal waves (Rapaport et al., 2004). In the human retina, RGCs are born first, followed by horizontal cells, cones, amacrine cells, rods, bipolar cells, and finally Müller glia (Cepko, 2014; Lu et al., 2020) (Figure 4). These cells are specified in two temporal waves or phases (Rapaport et al., 2004). During the first phase, retinal ganglion cells, horizontal cells, and cones are born. During the later phase, rods, bipolar cells, and Müller glia are born (Rapaport et al., 2004). Amacrine cells span the gap between phases (Rapaport et al., 2004). As mentioned, photoreceptors are divided between these waves, with cones being generated before rods (Carter-Dawson and LaVail, 1979; La Vail et al., 1991).

Photoreceptor differentiation occurs across the retina from the central fovea to the periphery in spatiotemporal waves of differentiation (La Vail et al., 1991; Xiao and Hendrickson, 2000). The specification of photoreceptors is determined by the expression of their respective opsin protein. S cones are specified first (as defined by S-opsin expression), followed by M/L cones (M/L-opsin expression) and finally, rods (rhodopsin expression) (Cepko, 2014; Eldred et al., 2018). At birth, some rods do not yet express rhodopsin, suggesting that rod generation and maturation continues postnatally (Hendrickson et al., 2008).

The genetic network specifying photoreceptor fate is well-studied. The transcription factor paired box 6 (PAX6) is expressed in multipotent progenitor cells and is essential for the specification of retinal cell fates (Marquardt et al., 2001). The receptor Notch is expressed in retinal progenitors to maintain progenitor state (Nelson et al., 2007). As progenitor cells exit the cell cycle and differentiate into photoreceptor fates, Notch expression ceases and cells express the orthodenticle homeobox 2 (OTX2) transcription factor (Jadhav et al., 2006; Muranishi et al., 2011). OTX2 is essential for photoreceptor fate specification and its absence leads to an amacrine-like fate (Nishida et al., 2003). OTX2 activates downstream genes including visual system homeobox 2 (VSX2) (Kim et al., 2008), PR/SET domain 1 (PRDM1) (Brzezinski et al., 2010; Katoh et al., 2010), and cone-rod homeobox protein (CRX) (Nishida et al., 2003). VSX2 is upregulated in bipolar cells (Kim et al., 2008) and represses rod photoreceptor genes (Dorval et al., 2006; Livne-Bar et al., 2006). PRDM1 stabilizes photoreceptor fate and prevents immature cells from differentiating into bipolar cells (Brzezinski et al., 2010) by repressing VSX2 (Katoh et al., 2010). As PRDM1 levels increase, it negatively feeds back onto OTX2, ultimately turning off its own expression and driving rod fate (Wang et al., 2014). Finally, CRX is present in all photoreceptors (Chen et al., 1997). CRX is important for photoreceptor maintenance, as CRX mutations result in cone-rod dystrophy (Freund et al., 1997) or Leber’s congenital amaurosis (Swaroop et al., 1999), which causes photoreceptor degeneration and vision loss. Conversely, overexpression of CRX increases rods at the expense of Müller glia and amacrine cells (Furukawa et al., 1997).

### Rod Specification

Upon acquiring photoreceptor fate, photoreceptors choose between cone and rod fates. RAR-related orphan receptor B (RORB) is a nuclear hormone receptor expressed in all photoreceptors. RORB acts upstream of Neural retina-specific leucine zipper protein (NRL), a critical regulator of rod fate (Jia et al., 2009; Kautzmann et al., 2011; Fu et al., 2014). NRL is necessary and sufficient for rod fate, as its loss results in a complete absence of rods and an increase in S cone-like photoreceptors (Mears et al., 2001; Akimoto et al., 2006), and ectopic expression leads to a loss of cones and an increase in rods (Oh et al., 2007). NRL directly binds and represses the promoters of both thyroid hormone receptor beta (THRB) and S-opsin, genes essential for cone specification (Oh et al., 2007). NRL also activates nuclear receptor subfamily 2 group E member 3 (NR2E3), which represses cone fate and activates a subset of rod genes including rhodopsin (Chen et al., 2005; Peng et al., 2005; Cheng et al., 2006; Oh et al., 2008). NR2E3 mutations result in improper lamination and enhanced S cone syndrome, with an increase in S cones and a number of visual defects (Haider et al., 2000; Jacobson et al., 2004).

Retinoic acid (RA) has also been implicated in the promotion of rod fate. In zebrafish, exogenous RA causes an increase in rods with a loss of cones (Hyatt et al., 1996). RA drives rhodopsin expression in cultured cells (Kelley et al., 1994; Kelley et al., 1999) and NRL expression in retinoblastoma cells and primary cell culture (Khanna et al., 2006). Regulation of NRL by RA may be direct as the NRL promoter contains a retinoic acid response element (RARE) (Khanna et al., 2006). Along with these regulators, taurine and sonic hedgehog (SHH) also promote rod fate in cultured cells (Altshuler et al., 1993; Levine et al., 1997).

### Cone Specification

Like rods, cones are specified by a distinct regulatory logic. One cut homeobox 1 (ONECUT1), a member of the Cut homeobox family of transcription factors, acts with OTX2 to promote cone fate by activating expression of nuclear thyroid hormone receptor THRB/B2, an early marker of cone fate (Sjoberg et al., 1992; Ng et al., 2009; Emerson et al., 2013). Discriminating the roles of THRB (including THRB1 and THRB2) versus the THRB2 isoform in human and mouse has been complicated (Ng et al., 2001; Roberts et al., 2006; Applebury et al., 2007; Weiss et al., 2012; Eldred et al., 2018), as *THRB2* lies interior in the *THRB* locus. THRB is a thyroid hormone receptor that acts as a transcription factor at thyroid response elements (TREs). In the absence of thyroid hormone, THRB is bound by corepressors. When thyroid hormone is present, it binds THRB, which induces the replacement of corepressors by coactivators, ultimately regulating gene expression (McNerney and Johnston, 2021). Thyroid hormone receptors can homodimerize or heterodimerize with retinoid X receptor gamma (RXRG), a nuclear receptor expressed in cones (Mori et al., 2001), to regulate aspects of cone subtype specification (Roberts et al., 2005). In mouse, heterodimers of RXRG and THRB play a role in establishing the S-cone pattern. Since the expression dynamics of these transcription factors are similar in human and mouse retinal development, this heterodimer pair might play a similar role in human cone patterning (Roberts et al., 2005). Additionally, POU2F2 has been shown in mouse to repress NRL and promote cone fate (Javed et al., 2020).

### Cone Subtype Specification: S Versus M/L Cone Fates

Human cones choose one of three subtype fates: S/blue, M/green, or L/red. Cone subtypes are specified in a two-step process. In the first decision, cones choose either the S or M/L cone fate. The second decision occurs between M and L cone fates.

Thyroid hormone signaling regulates the first choice between S and M/L cone fates. Thyroid hormone has two main forms: the active form, T3, which binds with high affinity to thyroid hormone receptors, and T4, the less active, circulating form (Samuels et al., 1974; Schroeder and Privalsky, 2014; McNerney and Johnston, 2021). Thyroid hormones cannot diffuse freely across cell membranes but rather require transporters such as MCT8 (Dumitrescu et al., 2004; Friesema et al., 2004). Moreover, deiodinase enzymes locally modulate levels of T3 and T4. Specifically, deiodinase 3 (DIO3) degrades both T3 and T4, while deiodinase 2 (DIO2) converts T4 into the active T3 (Dentice et al., 2013). DIO3 and DIO2 are expressed dynamically in the developing human retina as well as in the retinas of other species (Trimarchi et al., 2008; Ng et al., 2010; Guo et al., 2014; Bagci et al., 2015).

Foundational work in model organisms established a role for thyroid hormone signaling in cone subtype specification (Sjoberg et al., 1992; Ng et al., 2001; Roberts et al., 2006; Applebury et al., 2007; Trimarchi et al., 2008; Suzuki et al., 2013). Mice lacking THRB2 show a complete loss of M cones and only express S-opsin in cones (Ng et al., 2001; Roberts et al., 2006). Similarly, THRB knockouts display altered cone subtype ratios in fish (Suzuki et al., 2013; Mackin et al., 2019). Thyroid hormone signaling is also sufficient to regulate cone fates, as increasing T3 induces M-opsin expression in mice (Roberts et al., 2006; Glaschke et al., 2010; Glaschke et al., 2011). Moreover, knockdown of MCT8, a thyroid hormone transporter, altered cone subtype ratios in chicken (Vancamp et al., 2019).

Regulators of the thyroid hormone pathway are expressed in waves during development of the chicken retina. During early neurogenesis, a wave of *DIO3* expression in cone progenitors occurs from the center to the periphery (Trimarchi et al., 2008). As *DIO3* expression fades, *TRß* expression initiates, but is limited to a subset of progenitor cells (Trimarchi et al., 2008). A wave of *DIO2* expression in a subset of progenitor cells in the outer neuroblastic layer, an early, proliferative region of the retina, marks the beginning of photoreceptor differentiation, beginning as a ventral to dorsal gradient that gradually becomes restricted to the ventral retina. Simultaneously, a horizontal stripe of *DIO3* expression occurs across the central retina (Trimarchi et al., 2008). Over time, this *DIO3* expression expands to the periphery (Trimarchi et al., 2008). Lastly, *DIO3* expression is lost in a wave from the center to the periphery, concurrent with progenitor cell differentiation into Müller glia (Trimarchi et al., 2008). Following these waves of expression, *DIO3* is expressed in progenitors, *TRα* (another thyroid hormone receptor) is expressed in most cells of the retina, and *DIO2* is expressed in a subset of photoreceptors (Trimarchi et al., 2008). Together, these studies across multiple model organisms show that thyroid hormone signaling is spatially and temporally dynamic, and fundamental for retina development and photoreceptor differentiation.

Consistent with these studies in model organisms, clinical evidence supports a role for thyroid hormone signaling in cone subtype differentiation in the human retina. Premature infants with low ratios of T3 to T4 have an increased incidence of color vision deficiencies (Dowdeswell et al., 1995; Rovet and Simic, 2008; Simic et al., 2010; Yassin et al., 2019). Additionally, patients with mutations in *THRB* display altered color perception (Weiss et al., 2012). More recently, *DIO2* was found to be expressed in retinal precursor cells in the human macula during development (Lu et al., 2020). These studies implicate thyroid hormone signaling in human cone subtype specification.

To directly address the role of thyroid hormone signaling in human cone subtype specification, we studied human retinal organoids. Human retinal organoids are model retinas that are differentiated from human stem cells and grown in a dish. They recapitulate the temporal dynamics of human cone subtype specification during fetal development (Nakano et al., 2012; Zhong et al., 2014; Kaewkhaw et al., 2015; Wahlin et al., 2017; Eldred et al., 2018; Phillips et al., 2018). In human retinas and organoids, S cones are generated before M/L cones. *THRB* mutant organoids have S cones only, whereas addition of T3 in wild type organoids produces M/L cone-rich organoids. Moreover, the addition of T3 to *THRB* mutant organoids generates organoids with all S cones, indicating that T3 acts through THRB to specify M/L cones (Eldred et al., 2018). Thus, thyroid hormone signaling is necessary and sufficient to promote M/L cone fates and suppress S cone fates in humans.

The expression of thyroid hormone regulators in human retinas and retinal organoids is consistent with a temporal role of thyroid hormone signaling in the generation of S cones before M/L cones during human retinal development. DIO3, the enzyme which degrades T3, is expressed early, around the time when S cones are specified (Hoshino et al., 2017; Eldred et al., 2018). In contrast, DIO2, which synthesizes T3, is expressed later in development, when M/L cones are specified (Hoshino et al., 2017; Eldred et al., 2018). These studies suggest that expression of thyroid hormone regulators is temporally regulated to decrease thyroid hormone signaling early to specify S cones and increase signaling late to specify M/L cones.

Along with the role of thyroid hormone, PIAS3 is activated by TRB2 and RXRY in mice. PIAS3, as a result, is expressed at higher levels in M cones over S cones. PIAS3 then acts in M cones to SUMOylate RORA to repress S-opsin. PIAS3 can also act to enhance the effect of T3 driven M-opsin expression (Onishi et al., 2010). Interestingly, PIAS3-dependent SUMOylation of NR2E3 within rod cells can strongly prevent the expression of S cone genes (Onishi et al., 2009), suggesting multiple distinct roles for PIAS3 in retinal cell type specification.

The COUP family of transcription factors also plays a role in the specification of cone subtype specification in mice. Mice have a gradient of cone subtypes with more M cones in the dorsal retina and more S cones in the ventral retina (Wikler et al., 1996; Eldred et al., 2020). COUP-TFI suppresses M-opsin ventrally while COUP-TFI and COUP-TFII suppress S-opsin dorsally. In human cell lines, COUP-TFs repress S-opsin expression, suggesting these two transcription factors also play a crucial role in cone subtype specification (Satoh et al., 2009).

SALL3 also plays an important role in cone subtype specification. SALL3 is expressed in the S cones of mice and drives expression of S-opsin. SALL3 null mice showed a downregulation of S-opsin and phototransduction genes (de Melo et al., 2011). Though PIAS3, the COUP transcription factors, and SALL3 have not been directly implicated in human cone subtype specification, they are promising candidates to evaluate for roles through genetic manipulation in human retinal organoids.

### Cone Subtype Specification and Differences: M Versus L Cone Fates

Cones that adopt the M/L cone fate then choose between M or L cone fates. The only known difference between M and L cones is the expression of their respective opsin photopigment (Yamaguchi et al., 1997; Hagstrom et al., 2000; Peng et al., 2019). The M-opsin and L-opsin genes are located in a tandem array on the distal portion of the q arm of the X chromosome (Francois, 1958; Nathans et al., 1986a; Vollrath et al., 1988; Feil et al., 1990). As a result, regulation of these genes occurs at only one locus due to hemizygosity in males and X-inactivation in females (Wang et al., 1992).

The M- and L-opsin genes are regulated by a shared DNA element called the locus control region (LCR), which is required for the expression of both genes (Nathans et al., 1989; Wang et al., 1992). The LCR is an ancient promoter element that predates mammals with ancestral origins in fish (Tsujimura et al., 2007). Within mammals, the LCR is highly conserved among diverse species including humans, cows, and mice, and predates the duplication of M-opsin to generate L-opsin (Wang et al., 1992), yet the promoters of the opsin genes are not conserved.

The number and arrangement of M- and L-opsin gene copies relative to the LCR is variable. In the most common arrangement, the L-opsin gene is proximal to the LCR, followed by multiple copies of the M-opsin gene. M-opsin genes are most often found in a range of 1–5 copies with a mode of 2 (Drummond-Borg et al., 1989). No matter the number of M-opsin genes in the array, only the first M-opsin is expressed (Jorgensen et al., 1990; Winderickx et al., 1992; Yamaguchi et al., 1997; Hayashi et al., 1999). In addition to the proximal copy of the L-opsin gene, L-opsin genes are also sometimes observed in the distal array (Ueyama et al., 2015).

Two nonexclusive models for M and L cone specification have been proposed: the “stochastic” model and the “temporal” model (Smallwood et al., 2002). In the stochastic model, M and L cones randomly choose between these two fates. In this model, the regulatory LCR DNA element loops to either the M-opsin promoter or the L-opsin promoter to stably and exclusively drive expression of one opsin (Wang et al., 1999; Smallwood et al., 2002). This model is supported by transgene experiments in mice suggesting that the order and proximity of the opsin genes relative to the LCR controls the probability of opsin expression (Smallwood et al., 2002). These experiments suggest that the distal M promoter interacts with the LCR more effectively than the proximal L promoter.

In the temporal model, also known as the standard model, M cones are generated before L cones. Functional microspectrophotometry, multifocal-ERG imaging, and mRNA expression studies suggest that the retinal periphery has a higher proportion of L cones to M cones (La Vail et al., 1991; Mollon and Bowmaker, 1992; Hagstrom et al., 1998; Bumsted and Hendrickson, 1999; Martin et al., 2000; Otake et al., 2000; Xiao and Hendrickson, 2000; Roorda et al., 2001; Carroll et al., 2002; Bowmaker et al., 2003; Cornish et al., 2004; Neitz et al., 2006; Kuchenbecker et al., 2008). Since the retina develops from the center to the periphery, the earliest cells lie nearest to the fovea and the last-born cells are in the periphery. The higher proportion of L cones in the periphery suggest that L cones are the last cells to differentiate. Our analysis of M- and L-opsin expression suggested that M-opsin is expressed first in human fetal development, consistent with temporal regulation of the M/L cone fate decision (Hadyniak et al., 2021).

Experimental interrogation of the mechanism controlling M versus L cone subtype specification has been limited in large part due to the difficulty differentiating between the two cone subtypes. As the proteins are too similar to distinguish by antibodies, sequencing experiments and adaptive optics have been the primary methods used to understand the ratios of these two cone subtypes (Hofer et al., 2005). We recently took advantage of new advances in RNA *in situ* hybridization to directly visualize M and L cones and conduct spatiotemporal analysis of cone development (Hadyniak et al., 2021). This RNA *in situ* hybridization approach relies on using two partially overlapping oligos with three key nucleotide differences that enable binding specifically to the M- or L-opsin mRNAs.

Experimentally tractable model systems to study M versus L cone specification are lacking as the L cone subtype is unique to humans and non-human primates amongst placental mammals. Human retinal organoids provide a promising model to test mechanisms of human-specific developmental mechanisms. Treatment of retinal organoids with exogenous retinoic acid during early stages of development promotes M cone fate at the expense of L cones (Hadyniak et al., 2021), providing the first cue to a pathway responsible for the differences in M and L cone fates.

### Variation in the Ratios of Cone Subtypes

The percentage of S cones (8–12%) relative to M/L cones in the human retina is consistent (de Monasterio et al., 1985; Ahnelt et al., 1987; Szél et al., 1988; Lerea et al., 1989; Ahnelt et al., 1990; Nork et al., 1990; Curcio et al., 1991). In contrast, the ratio of L:M cones (historically reported as L before M) in the human retina varies dramatically across the population from 1:4 to 16.5:1, with an average ratio of 2:1 (De Vries, 1946; Rushton and Baker, 1964; Dartnall et al., 1983; Cicerone and Nerger, 1989; Jacobs and Neitz, 1993; Yamaguchi et al., 1997; Hagstrom et al., 1998; Brainard et al., 1999; Roorda and Williams, 1999; Deeb et al., 2000; Kremers et al., 2000; Otake et al., 2000; Carroll et al., 2002; Hofer et al., 2005; McMahon et al., 2008). The range of variability in L:M cones implicates a level of biological plasticity as individuals who have extreme L:M ratios retain normal color vision.

Evidence suggests that changes in the nucleotide sequences in the L/M-opsin loci are associated with variation in the ratio (Gunther et al., 2008), but the differences between the L and M promoters did not account for this variation (McMahon et al., 2004). Our studies identified changes in the non-coding antisense RNA of NR2F2, a transcription factor activated by retinoic acid, that are associated with changes in the L:M ratio, consistent with the role for RA and its sufficiency to generate M cones early in retinal organoids (Hadyniak et al., 2021).

In contrast to the highly variable L:M ratio in humans, the ratio in non-human primates is more reproducible and more or less equal, ranging from 0.6:1 to 1.17:1 (Marc and Sperling, 1977; Mollon and Bowmaker, 1992; Packer et al., 1996), suggesting evolutionary differences in mechanisms of L and M cone differentiation between primates.

Technical challenges may complicate the analyses of M and L cone ratios. As M and L cones could not be visualized directly in fixed tissue prior to our recent studies, measurement methods may contribute to the variability observed in humans. Methods that rely on mRNA copy number assume equal transcription between M and L cone opsin genes. Physiological methods assume that each M and L cone contributes equally to the visual response. Microspectrophotometry, using a combination of high-resolution retina imagining with retinal densitometry, provides one of the most accurate forms of individual visualization of the L/M cone ratio (Hofer et al., 2005). Our development of an *in situ* hybridization approach to distinguish *M-* and *L-opsin* will enable new studies of variation in fixed tissue with single cell resolution moving forward (Hadyniak et al., 2021).

### Photoreceptor Developmental Disorders

Mutations in essential rod pathway genes can lead to visual disorders. NR2E3 is a transcription factor that drives rod fate, including rhodopsin gene expression (Cheng et al., 2004; Peng et al., 2005; Cheng et al., 2006). Patients with mutations in *NR2E3* can present with enhanced S cone syndrome (a condition in which the retina lacks rods but produces an unusually high number of S cones), night blindness, hypersensitivity of S cones, impairment of M and L cones, loss of rod function, and a decrease in total rods (Jacobson et al., 1990; Marmor et al., 1990; Hood et al., 1995). One study identified a loss of rods along with double the typical number of cones, with 92% of those being S cones (Milam et al., 2002). These clinical data suggest that this gene is responsible for suppressing the S cone fate and driving proper rod specification (Haider et al., 2000; Haider et al., 2006).

Similarly, mutations in cone specification and development leads to human visual disorders. Color blindness affects 1 in 12 Caucasian males and 1 in 200 Caucasian females (McKusick, 2007). Mutations which cause cone-related disease and color blindness can come from essential cone genes or from mutations in the cone opsins themselves. The most severe form of color blindness is achromatopsia, marked by the true absence of color discrimination and monochromatic color vision. The two main types of achromatopsia are rod monochromacy and S cone monochromacy. Rod monochromacy is an autosomal recessive disease (Spivey, 1965; Fleischman and O'Donnell, 1981) in which individuals have normal rods with functioning rhodopsin, but no cone sensitivity (Sloan, 1954; Blackwell and Blackwell, 1961). This is typically caused by mutations in essential cone genes such as activating transcription factor 6 (ATF6), cyclic nucleotide-gated cation channel alpha-3 (CNGA3), cyclic nucleotide-gated channel subunit beta-3 (CNGB3), G protein subunit alpha transducin 2 (GNAT2), or phosphodiesterase 6C (PDE6C) (Rosenberg et al., 2004; Vincent et al., 2011; Kohl et al., 2012; Carss et al., 2017; Skorczyk-Werner et al., 2017). S cone monochromacy is an X-linked disease in which individuals have rods and S cones, but completely lack M and L cones (Blackwell and Blackwell, 1961; Alpern et al., 1965; Pokorny et al., 1970; Alpern et al., 1971; Daw and Enoch, 1973; Alpern and Pugh, 1974; Smith et al., 1978; 1979).

Another form of color blindness, dichromacy, is marked by the absence or loss of function of one cone subtype. Loss of expression of a cone subtype-specific opsin is linked to the loss of function of the respective cone subtype. The most common form of color blindness in the population is anomalous trichromacy, where individuals have altered spectral sensitivity of one opsin. Anomalous trichromacy is caused by point mutations in opsin genes or gene rearrangements (Nathans et al., 1986a).

In addition to these disorders caused by breakdowns in specification or function during development, numerous degenerative diseases affecting rods and cones cause vision loss [reviewed in (Sancho-Pelluz et al., 2008; Liu et al., 2010; Wright et al., 2010)].

## Patterning of Photoreceptors Across the Human Retina

We now shift our discussion from the mechanisms that specify photoreceptor subtypes to their patterning in the human retina. Foundational work, particularly in the 1980s through the 2000s, informed our current knowledge of the spatiotemporal patterning of human photoreceptors during development. Rods outnumber cones 20:1 across the retina, but this ratio varies in different regions, with 1:1 near the center and 30:1 in the periphery (Curcio et al., 1990). The human retina is patterned into discrete, concentric regions, each with unique compositions of photoreceptor subtypes at distinct densities. Additionally, there are some local patterns of photoreceptor patterning. We first discuss local patterning, then move on to photoreceptor patterning in concentric regions, and finally discuss axial patterning in the nasal/temporal and superior/inferior axes.

### Local Patterning

The adult human retina has local patterning of rods and cones. Cones are hexagonally patterned near the central fovea, and as eccentricity increases, rod populations increase and disorder the cone hexagonal pattern (Sawides et al., 2017). Rod density varies with distance from the central fovea. Around 500 μm from the fovea, rods begin to encircle cone cells (Curcio et al., 1990).

Local patterning also occurs for cone subtypes. At a distance of ∼0.5 mm from the fovea, S cones are randomly patterned (Roorda and Williams, 1999; Roorda et al., 2001). At an eccentricity of 3 mm, however, the S cone distribution becomes more regular (Curcio et al., 1991; Bumsted and Hendrickson, 1999; Martin et al., 2000). The local arrangement of M and L cones is proposed to be random in the human retina (Roorda and Williams, 1999; Otake et al., 2000; Roorda et al., 2001; Bowmaker et al., 2003), based on studies using microspectrophotometry and retinal densitometry combined with adaptive optics (Mollon and Bowmaker, 1992). However, other analysis suggests nonrandom clumping of M cones, supporting elements of regulated patterning (Roorda et al., 2001).

### Concentric Patterning

**FIGURE 5:**
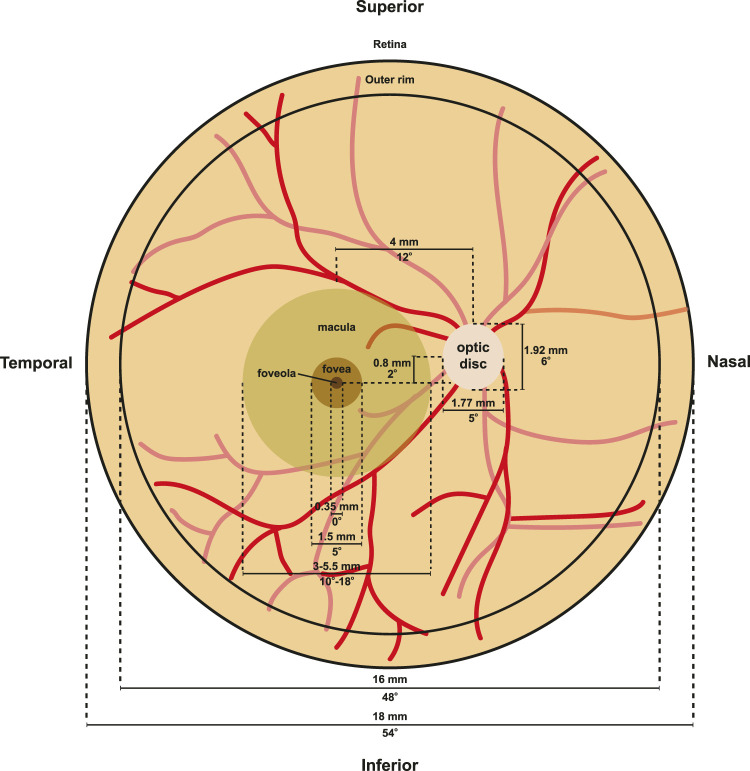
Retina morphology. Flat mount diagram of a human retina. Optic disc measurements were derived from (Arora et al., 2015), while macula, fovea, foveola, and outer rim measurements from (Schultze, 1866; Polyak, 1941; Curcio et al., 1990; Curcio et al., 1991; Hendrickson, 1992). Red lines indicate vasculature.

The patterning of photoreceptors across the human retina can be divided into several concentric rings with distinct photoreceptor constituencies and morphologies. From the periphery to the center, the retina contains the peripheral rim, posterior pole, macula, fovea, and foveola (Figure 5). Here, we discuss the differences in photoreceptor patterning and function in these regions:

### Periphery and Outer Rim

The peripheral retina makes up the majority of the retina (Figure 5). At eccentricities from 5 to 16 mm, cones decrease in density, reaching a low of about 2000 cones/mm^2^ (Farber et al., 1985; Curcio et al., 1990; Jonas et al., 1992). Rods, in contrast, increase to around 6,500 rods/mm^2^ outside the macula from 5 to 8 mm, and then decrease to 58,000 rods/mm^2^ from 8–16 mm (Curcio et al., 1990; Jonas et al., 1992). At the edge of the retina at 16–18 mm from the fovea center, cone density increases to 5,000 to 7,000 cones/mm^2^ (Curcio et al., 1990). At the outer rim, a 1 mm band surrounding the edge of the retina, cone density increases three-fold and rod density decreases 10-fold (Williams, 1991).

Peak rod density in the periphery appears important for low-light vision and motion detection. Though the periphery is over 90% of the retina, it does not play a substantial role in high-acuity daytime vision (Curcio et al., 1990; Quinn et al., 2019). The functional role of the cone-rich outer rim is poorly understood.

### The Macula

Collectively known as the macula, the central retina is responsible for high acuity vision and perception of color in humans (Figure 5). Visual acuity is much lower in the peripheral macula compared to the central macula (fovea). The macula is 3–5.5 mm in diameter and is located approximately 4.7 mm temporal from the optic nerve (Jonas et al., 2015). The macula is characterized by a yellowish hue known as the macular pigment (Delori et al., 2001). The macular pigment contains a number of carotenoid pigment molecules including zeaxanthin, meso-zeaxanthin, and lutein (Bone et al., 1985), which limits chromatic blur to increase visual acuity (Reading and Weale, 1974) and protects the macula from photodegradation by short wavelength light (Kirschfeld, 1982).

Despite making up just 4% of the retinal surface area, the macula contains a majority of the cones and RGCs in the retina (Curcio and Allen, 1990). All three subtypes of cones are present at high density within the macula. From the central rod-free foveola, rods are introduced into this mosaic (Curcio et al., 1991). Rods quickly outnumber cones in the macula, reaching a ratio of 4:1 at approximately 0.66 mm from the center of the foveola (Curcio et al., 1990), and ending at a ratio of 8:1 throughout the macula (Curcio et al., 1990). In addition, the macula contains the largest portion of RGCs in the retina. The central retinal RGCs project to a larger proportion of the visual cortex compared to the RGCS of the peripheral retina (Tootell et al., 1988; Curcio et al., 1990).

The macula is particularly susceptible to degeneration later in life. This could be related to the high rate of metabolic stress in the region as a result of the high density of photoreceptors and retinal ganglion cells. In addition to the roles that Müller glia play in protecting the fovea from blue light and acting as optical fibers, they likely also protect the neurons, including the photoreceptors, of the retina from oxidative stress, along with supporting neuronal homeostasis and survival. Compared to peripheral Müller glia, macular Müller glia show increased expression of phosphoglycerate dehydrogenase, an important enzyme in the serine synthesis pathway (Zhang et al., 2019). When phosphoglycerate dehydrogenase was inhibited in cultured macular Müller glia, they were more susceptible to oxidative stress (Zhang et al., 2019). Additionally, Müller glia have recently been shown in mice to play a role in serine biosynthesis, which can prevent photoreceptor degeneration observed in phosphoglycerate dehydrogenase deficiencies (Shen et al., 2021). These experiments suggest that Müller glia play a critical role in the health and maintenance of the photoreceptors of the human macula.

### The Fovea

The fovea is the specialized region at the center of the macula that limits light scattering and increases visual acuity (Figure 5). The fovea is located approximately 4 mm temporal and 0.8 mm inferior to the optic nerve head (Hogan et al., 1971). The fovea is about 1.0–1.5 mm wide and 200–240 μm deep, which is approximately half the thickness of the surrounding retina (Polyak, 1941), though these numbers can vary between individuals (Figure 5).

The high acuity of the fovea, including the central foveola, is enabled by the high cone density, the ratio of cones to bipolar cells to RGCs, and cortical magnification. Cortical magnification describes the process whereby the central fovea, which makes up only 0.01% of the area of the human retina, maps to 8–10% of the visual cortex (Goldstein, 2010). In comparison, the central 5 degrees of the retina (which also encompasses the foveal pit), maps to approximately 40% of the visual cortex (Tootell et al., 1988; Curcio et al., 1990). To utilize these visual adaptations, the human eyes are constantly moving such that the binocular foveas are focused on the object of interest (Goldberg, 2014).

Foveal L, M, and S cones are dense and morphologically distinct. In the fovea, cones have smaller inner and outer segment diameters compared to cones in the rest of the retina (Farber et al., 1985), Cones have an inner segment diameter of 1.6–2.2 μm in the fovea (Curcio et al., 1990) versus 6–8 μm in the periphery (Liu et al., 2018). Cones in the fovea are tightly packed in a triangular lattice.

The fovea contains the highest density of the ∼4 to 5 million cones in the retina in only 0.02% of the area and a large proportion of the total retinal ganglion cells (Curcio and Allen, 1990; Curcio et al., 1990). The peak cone density in the central fovea is ∼200,000 cones/mm^2^, but can range from 100,000 to 325,000 cones/mm^2^ (Curcio et al., 1990). This density decreases by an order of magnitude to 20,000 cones/mm^2^ towards the edge of the fovea at 1 mm from the central foveola. This decrease is more significant along the vertical axis than the horizontal axis, consistent with the presence of a cone streak along the horizontal meridian (Packer et al., 1989; Curcio et al., 1990). In contrast, rods appear at 100–200 μm from the foveal center and S cones peak at 15% of the total cone ratio at 200–300 μm (de Monasterio et al., 1985; Szel et al., 1988; Curcio et al., 1991; Cornish et al., 2004). Throughout the rest of the retina, S cones are present at 8–10% of the cone population. Rod density increases until rods and cones are equal in number at 400–500 μm from the foveal center. As rods enter the mosaic, cones lose their triangular lattice packing.

In addition to these photoreceptor patterns, several morphological features enhance visual acuity in the human fovea. The fovea is concaviclivate, or dish-shaped, with displacement of the majority of the retinal layers except for the cone photoreceptors (Figure 1). This displacement of inner retinal layers and absence of vasculature limits light scattering and enhances photon absorption efficiency by the outer segments of photoreceptors (Walls, 1937; Polyak et al., 1957). Chromatic aberration is also reduced by Müller glia, which act as fibers to guide light to the cones in the fovea (Franze et al., 2007). The physical shape of the foveal pit places Müller glia on top of cones, forming a cone of Müller glia on top of the foveal cone cells in the space normally occupied by the inner retinal layers (Yamada, 1969). Patients with foveal hypoplasia, a disease in which pit formation does not occur, present with reduced visual acuity, suggesting a role in pit formation for proper visual acuity in humans (Thomas et al., 2011; Wilk et al., 2014; McCafferty et al., 2015).

Differences in retinal circuitry also enhance visual acuity in the fovea. In the fovea, cones signal via elongated axons (i.e. fibers of Henle) to centrifugally displaced bipolar cells at the walls of the fovea. A single foveal M or L cone signals to two midget bipolar cells (an ON and an OFF). This signal is then transmitted to a corresponding ON or OFF midget ganglion cell such that each RGC receives input from one M or L cone, forming a “private” line (Kolb and Marshak, 2003). In the fovea, the ratio of cones to RGCs is 1:2 or 1:1 (Wassle et al., 1989; Curcio et al., 1990) consistent with a midget system of 1 cone: 2 bipolar cells: 2 RGCs (Drasdo et al., 2007). However, some RGC types may receive information from more than one cone, consistent with a measurement of 3.34 RGCs per 1 cone in the fovea (Wassle et al., 1989). The midget circuit is best characterized for M/L cones, though an S cone specific pathway has been identified in macaque (Klug et al., 2003; Patterson et al., 2019). Midget circuitry contrasts between the fovea and peripheral retina. In the peripheral retina, midget ganglion cells receive input from many more bipolar cells, and each bipolar cell receives input from multiple rod or cone photoreceptors, resulting in information from as many as 10–30 cones (Sinha et al., 2017). These midget systems allow for increased visual acuity by reducing noise as each photoreceptor has almost a direct line to an individual RGC, which transfers the information to a large visual processing system in the brain.

### The Foveola

At the central base of the foveal pit lies the foveola (Figure 5), an elliptical region with an average size of 0.35 mm in horizontal diameter along the length of the ellipse (Schultze, 1866; Polyak, 1941; Curcio et al., 1990; Curcio et al., 1991; Hendrickson, 1992) and a thickness of only 100 μm (Yamada, 1969; Burris et al., 2002) compared to over 300 μm in the thickest regions at the foveal edge (Myers et al., 2015). The foveola contains the highest density of photoreceptors in the retina, ranging from 50,000 cones/mm^2^ (Osterberg, 1935; Farber et al., 1985; Ahnelt et al., 1987) to 180,000 cones/mm^2^ (Jonas et al., 1992), suggesting a high degree of variability in foveal cone density. Changes and variability in foveola cone densities are not surprising as loss of photoreceptors within the macula region occurs with aging (Gartner and Henkind, 1981).

The foveola contains M and L cones, no S cones, and no rods (Willmer, 1944; Willmer and Wright, 1945; Wald, 1967; Williams et al., 1981). Less is known about the ratio of M to L cones in the fovea, due to the similarity between these opsin subtypes and the challenges in distinguishing them. Psychophysics experiments suggest that the ratio of L to M cones is 2:1 in the fovea and foveola (Cicerone and Nerger, 1989). Direct measurements of the human retina using adaptive optics suggests that the proportion can vary greatly (Roorda and Williams, 1999).

A zone 365 μm in diameter with few to no S cones is first observed at around fetal week 15 (Xiao and Hendrickson, 2000). In adults, a range of sizes for the S cone-free region has been described, from 35–40 μm (Wald, 1967; Nork et al., 1990) (Williams et al., 1981; Curcio et al., 1991) to 300 μm (König, 1894). The size of the S cone-free zone may vary between individuals, with sparse S cone patterning within the region in some individuals, but the techniques used to determine the S cone-free region may not be able to resolve smaller regions (Williams et al., 1981). Consistent with the possible patterning of S cones in the foveola, Ahnelt and colleagues identified 3–5% of foveal cones as S cones based on morphology (Ahnelt et al., 1987).

Like the S cone-free zone, the rod-free zone in the foveola and fovea appears to change during development. The rod-free zone decreases from over 1,600 μm in diameter at 22 weeks of gestation to 650 μm at 4 years of age (Yuodelis and Hendrickson, 1986). The final diameter of the rod free zone varies postnatally, ranging from 350 to 720 μm (Yuodelis and Hendrickson, 1986; Curcio et al., 1990). Together, these observations suggest that the S cone-free and rod-free zones of the foveola develop dynamically and are likely somewhat variable across the population.

To achieve high visual acuity, a large number of neurons are needed to transmit the signals from the many photoreceptors in the foveola. High densities of photoreceptors can connect at ratios near 1:2:2 to bipolar cells and retinal ganglion cells to signal visual information to the brain, but this can result in a local increase in retinal thickness, which is detrimental to visual acuity. The foveola is designed to simultaneously allow for an increase in cell density and direct excitation of photoreceptors by photons of light to enable increased visual acuity by decreasing light scatter through centrifugal displacement of downstream neurons (Walls, 1937; Polyak et al., 1957). In this way, light can directly hit the photoreceptors because the connecting neurons are pushed aside, generating the foveal pit. This displacement to limit light scattering along with the midget circuitry that allows for a near 1:1 relationship between photoreceptors and downstream targets, provides the human foveola with high acuity vision (Polyak, 1941; Jusuf et al., 2006).

### Axial Patterning

**FIGURE 6:**
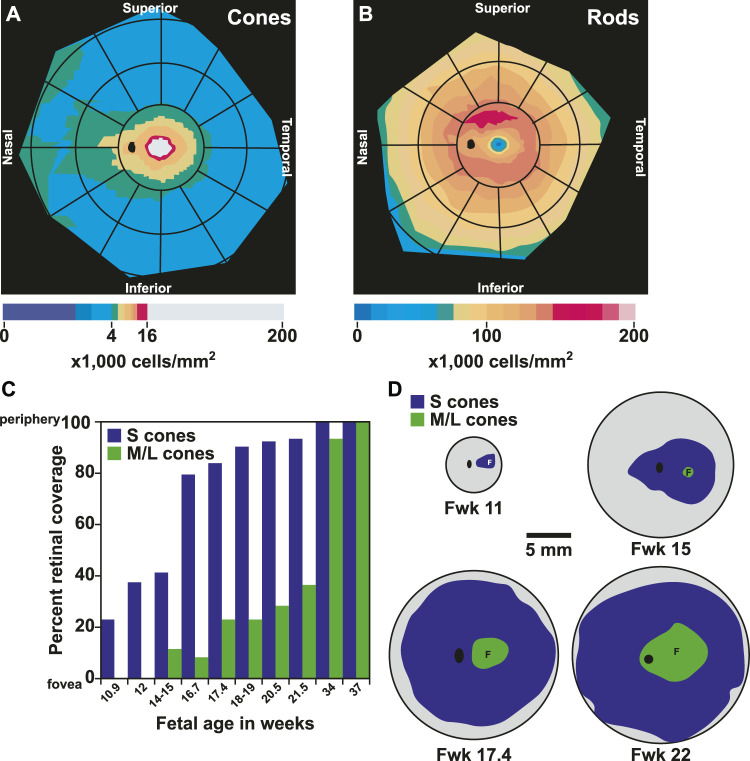
Patterning and migration of cones during development. **(A)** Heat map of cone density modeled from (Curcio et al., 1990). Cones range from 0 to 16,000 cells/mm^2^ at intervals of 1,000 cells/mm^2^. Densities above 16,000 cells/mm^2^ are represented by gray. **(B)** Heat map of rod density modeled from (Curcio et al., 1990). Rods range from 0 to 200,000 cells/mm^2^ at intervals of 12,500 cells/mm^2^ **(C)** Graph of fetal age and onset of S and M/L cones by percent retinal coverage, adapted from (Xiao and Hendrickson, 2000). **(D)** S and M/L specification by onset of opsin protein during fetal retinal development modeled from (Xiao and Hendrickson, 2000). Gray area is growing retinal tissue representing retinal progenitors. Fwk = fetal week. F = fovea center, black oval = optic disc.

The human retina has unique patterning of photoreceptors along the nasal-temporal and superior-inferior axes. Humans have a streak of high cone density found along the nasal-temporal axis at the horizontal meridian of the retina (Curcio et al., 1990) (Figure 6A). The nasal retina has up to 45% higher cone density compared to the temporal retina (Curcio et al., 1990).

In contrast to cone density, which is greatest in the central retina, rod density is greater and rod diameter is smaller in the temporal retina 5 mm from the center (Curcio et al., 1990). At 8 and 16 mm from the center, rods are less numerous and larger in the temporal retina compared to the nasal retina (Curcio et al., 1990) (Figure 6B). Along the superior-inferior axis, rods increase to 160,000–190,000 rods per mm^2^ in a hotspot in the superior retina. This hotspot occurs in an ovular ring at a similar distance from the foveal center as the optic disc (Curcio et al., 1991), and currently the purpose of the rod hotspot is not known. Rod density is lowest in the foveola as it passes the cone-rich horizontal meridian (Curcio et al., 1990). This scarcity of rods allows for the increased presence of cone cells to mediate visual acuity and color perception.

## Photoreceptor Patterning and Foveal Formation During Development

As we have described, the human retina is made up of many distinct, concentric regions comprised of unique populations and densities of photoreceptor subtypes. Development of this pattern of photoreceptor topography occurs in a sequence of waves of differentiation beginning at the foveola and spreading out into the peripheral retina over gestational time. Though cone differentiation begins in the fovea, this region is the last to fully develop. Pit formation and complete displacement of the inner retinal layers is not complete until 1 or 2 years of age. Additionally, foveal cone inner and outer segments continue to grow and the cones increase in density until around the age of 10, at which point this region resembles the adult (Hendrickson, 1992).

Photoreceptor differentiation starts at the foveola with the generation of cones. The differentiation of cones and then rods spreads out to the periphery (La Vail et al., 1991; Xiao and Hendrickson, 2000). S cones are specified first, followed by M/L cones, and finally by rods (Figures 6C,D) (La Vail et al., 1991; Xiao and Hendrickson, 2000). Some rods do not yet express rhodopsin at birth, suggesting that the process of maturation could continue postnatally (Hendrickson et al., 2008).

At 10–12 weeks of gestation, the first S opsin-expressing cones are observed in the presumptive fovea in the central retina (Hendrickson, 1992). After differentiation, cones form synapses in the fovea (Yuodelis and Hendrickson, 1986). By the 14th week of gestation, cell division has ceased in the fovea (Provis et al., 1985) and the wave of cones has reached the optic disc (Xiao and Hendrickson, 2000).

Rods are first observed as NRL-expressing cells around the fovea at fetal week 10.5–12 (Xiao and Hendrickson, 2000; Hendrickson et al., 2008), and express rhodopsin at fetal week 15 (Hendrickson et al., 2008). While the adult fovea is rod-free, the presumptive fovea in the fetal retina contains NRL- and/or rhodopsin-expressing photoreceptors that are lost by fetal week 20, suggesting that these immature rods are in some way eliminated from the fovea (Bumsted et al., 1997; Bumsted and Hendrickson, 1999; Xiao and Hendrickson, 2000; Hendrickson et al., 2008).

Foveal development appears to involve two series of neuronal migrations. The first migration occurs from fetal week 24 until 15 months after birth. During this time, bipolar cells, horizontal cells, amacrine cells, and RGCs migrate peripherally, promoting pit formation (Yuodelis and Hendrickson, 1986). In addition to this displacement, central migration of cones into the foveola increases packing. As these cones migrate centrally, they become narrower and more elongated, which allows for increased density. These processes may promote displacement of rods (Hendrickson and Yuodelis, 1984; Yuodelis and Hendrickson, 1986). Migration can be visualized through the fibers of Henle, the long cone axons that form synaptic connections with their partner bipolar cells prior to migration. These axons can reach lengths of up to 300 μm (Hollenberg and Spira, 1973; Smelser et al., 1974; Hendrickson and Kupfer, 1976). As the adult foveola contains only M and L cones, it is possible that M and L cones migrate internally past the S cones (Ahnelt et al., 1987; Curcio et al., 1991), though this seems unlikely as downstream synaptic connections have already been established and would result in tangled axons. Another possibility is that these S cones are eliminated in some yet unknown mechanism. Consistent with a role for cell migration in foveal photoreceptor patterning, the densities of cones in the fovea and rods in the region neighboring the fovea increase in the absence of local mitoses (Diaz-Araya and Provis, 1992). During these migration and packing events, the rod-free zone decreases from a diameter of 1,605 μm at 22 weeks gestation (Yuodelis and Hendrickson, 1986), to an average diameter of 350 μm by adulthood (Curcio et al., 1993).

The fovea is unique to primates among mammals, making experimental studies of development and photoreceptor patterning in this region challenging. Studies of the chicken retina, which contains a region analogous to the fovea called the high acuity area (HAA), suggest mechanisms controlling human foveal development. Similar to the human fovea, the HAA is cone-rich, rod-free, and surrounded by large populations of RGCs (da Silva and Cepko, 2017). In some avian species, HAAs have a pit, lack blood vasculature, and have a unique arrangement of interneurons known as the aster (da Silva and Cepko, 2017). Patterning of the HAA in chick depends on low retinoic acid (RA) signaling (da Silva and Cepko, 2017). The RA degrading enzymes CYP26A1 and CYP26C1 are highly expressed in the HAA, whereas the RA synthesizing enzymes RALDH1/ALDH1A1 and RALDH3/ALDH1A3 are repressed, consistent with RA degradation in this region (da Silva and Cepko, 2017). RA is sufficient to increase rods in the rod-free zone, decrease retinal ganglion cell density, and disrupt the aster in the inner nuclear layer of the high-acuity area (da Silva and Cepko, 2017). The patterning of RA regulatory enzymes is conserved in the developing human retina (da Silva and Cepko, 2017). Moreover, single cell RNA sequencing showed increased *CYP26A1* expression in the human fovea compared to the peripheral retina (Peng et al., 2019; Lu et al., 2020). These findings suggest that limiting RA signaling is also important for human foveal development.

## Future Directions: Human Retinal Organoids Provide a Model to Study Photoreceptor Development and Patterning

We are only beginning to understand how photoreceptors are specified in the human eye. Studies in model organisms have been critical in our understanding of retinal development, photoreceptor subtype differentiation, and retinal patterning of photoreceptors. Some mechanisms of photoreceptor differentiation, including specification of rods by NRL and cone subtypes by thyroid hormone signaling, are well-conserved between humans and other vertebrates.

The next challenges lie in understanding human-specific features of photoreceptor differentiation and patterning. The advent of human retinal organoids enables the generation of human retinal tissue in a dish using embryonic stem cells or induced pluripotent stem cells. Organoids develop on human timescales and follow the same temporal dynamics of cell type differentiation seen in the human retina, making them a powerful, emergent system to study mechanisms of human photoreceptor differentiation. First developed in 2012 by Yoshiki Sasai’s group (Nakano et al., 2012), human retinal organoids provide a tractable system to study and manipulate processes unique to the human retina. Since their development, a number of methods have emerged for generating retinal organoids from gravity aggregation to 2D/3D embryoid bodies to microfluidic chips with retinal cell types (Guy et al., 2021; Marcos et al., 2021).

Human organoids allow for genetic and pharmacological manipulations of human tissue, as well as observations of human development. Retinal organoids thus hold potential for better understanding basic human biology as well as developing therapeutics for retinal degenerative diseases and injuries. scRNA-seq studies confirmed that retinal organoids specify cell types in the same temporal order as the developing human retina (Clark et al., 2019; Cowan et al., 2020; Sridhar et al., 2020). Using this system, we showed that thyroid hormone plays an important role in the specification of S vs. M/L cones (Eldred et al., 2018) and that retinoic acid regulates the generation of M vs. L cones (Hadyniak et al., 2021). NRL null human retinal organoids display a lack of rods and an increase in S cones (Kallman et al., 2020). Recently, human retinal organoids have also been shown to be a useful model for studying cis-regulatory elements and their roles in disease and normal human development. Specifically, perturbation of an enhancer (5q14.3) involved in age-related macular degeneration among other diseases resulted in organoids that showed a delay in cell type specification and a partial loss of rods (Thomas et al., 2022). These types of mechanistic discoveries were not possible prior to the development of this *in vitro* system. Human retinal organoids are at the forefront of understanding human developmental biology and are sure to provide huge advances in our understanding of the human retina in the coming years.

One major challenge is that human retinal organoids do not recapitulate the concentric patterning of photoreceptors observed in humans. Additionally, human retinal organoids fail to form a fovea, preventing studies of pit formation, the mechanisms that yield high populations of M and L cones, cell displacement, and migration. One solution may lie in modulation of retinal organoid protocols to adjust the concentrations of thyroid hormone and retinoic acid, two factors that drive foveal-like cone patterning in other organisms. Additionally, tissue engineering could aid in mechanically forming a foveal-like pit in the organoid structure and introducing vascularization that enables better survivability and increased size. With larger organoids, local treatment of small molecules and hormones could be administered to create retinal organoids patterned more comparably to the adult retina. Currently, organoids remain too small to allow for such experiments, resulting in experiments that generate either more “foveal-like” or “peripheral-like” organoids, instead of organoids with a combination of specialized regions.

Non-human primates, especially rhesus macaques, provide an alternative model for retinal studies. They share similar patterning of photoreceptors as well as a high-acuity macula and fovea, and as such, are good models for understanding spatiotemporal development of photoreceptors and macular degeneration. Additionally, non-human primates are amenable to genetic manipulations including CRISPR (Kang et al., 2019), optogenetics (Chaffiol et al., 2017), adeno-associated virus (AAV) (Vandenberghe et al., 2013), and transplants (Picaud et al., 2019). This makes non-human primates a very promising model for studying primate-specific retinal features and patterns that organoids have not yet been optimized to examine.

Additional challenges come from the difficulty in differentiating M versus L cones. Recent advances in *in situ* technologies enabled us to differentiate between these two cone subtypes in fixed tissue at a single-cone resolution (Hadyniak et al., 2021). This new advance allows us to label cones in retinal organoids and study perturbations in development, along directly visualizing cone ratios in human samples in regions of the retina. With this new technology, we found that retinoic acid signaling regulates the M to L fate decision in retinal organoids (Hadyniak et al., 2021). Improvements to this technology will enable for visualization of M- and L-opsin expression with other genes of interest in multiple retinal cell types. These advances suggest that we will learn much more about the patterning and development of photoreceptors through experimentation in human retinal organoids.
